# Supplementary figures and images for: Hepatic autotaxin overexpression in infants with biliary atresia
Source: PeerJ. 2018 Jul 24;6:e5224. doi: 10.7717/peerj.5224 (PMC6063256; doi:10.7717/peerj.5224)

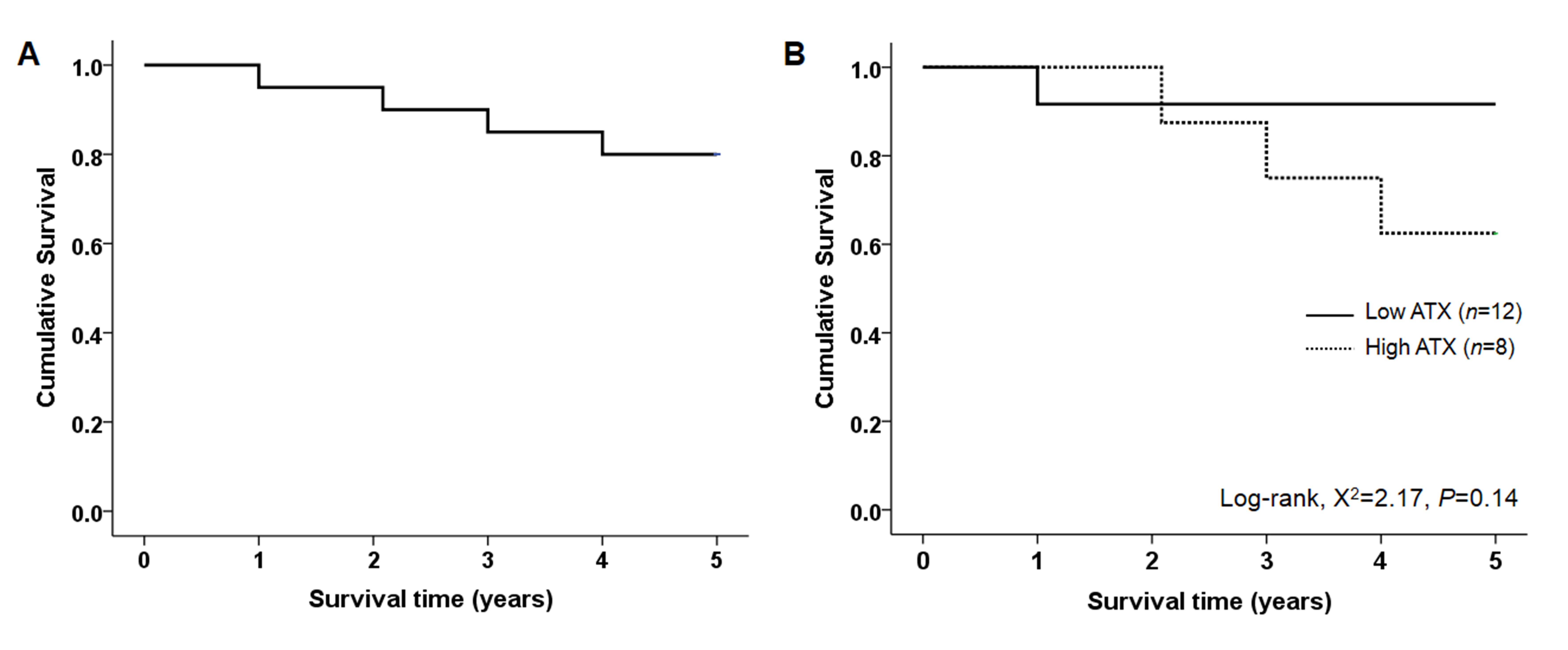

Supplement: Figure S1 — (A) The overall survival curve of 20 BA shows that 5-year survival rates with native livers are 80%. (B) Survival curve comparisons demonstrate that BA with low ATX expression (n = 12) have 5-year survival greater than those with high ATX expression (n = 8). [file peerj-06-5224-s002.png]
